# Precarization via Digitalization? Work Arrangements in the On-Demand Platform Economy in Hungary and Slovakia

**DOI:** 10.3389/fsoc.2020.00003

**Published:** 2020-02-26

**Authors:** Marta Kahancová, Tibor T. Meszmann, Mária Sedláková

**Affiliations:** Central European Labour Studies Institute, Bratislava, Slovakia

**Keywords:** precarity, digitalization, on-demand work, platform work, Central and Eastern Europe

## Abstract

The concept of precarity is increasingly used for an analysis of standard and non-standard (atypical) employment forms—yet among atypical employment forms, platform-driven work is rarely included. This paper aims to fill this gap and provide a refined analytical framework for an evaluation of precarity in employment arrangements applicable to on-demand platform work. The legitimacy of such an analytical framework is two-fold. First, it allows identifying the dimensions of precarity in on-demand platform work. Second, it extends the understanding of how a general situation in the labor market connects to work precarity in on-demand platform work. The analytical framework is applied to evidence from two countries in Central and Eastern Europe—Hungary and Slovakia, where the rise of precarious employment went hand in hand with the rise of work via digital platforms. The central claim of the paper is that precarity in on-demand platform work is especially manifest in the dimensions of autonomy at work and of interest representation. Furthermore, digitalization enforces precarity, while at the same time, it mitigates labor market segmentation between standard and non-standard workers as distinct groups of workers.

## Introduction

This paper seeks to establish a systematic conceptual and empirical relationship between two phenomena that recently sparked great research attention. The first one is the rise of non-standard work arrangements, some of which are considered precarious because of being uncertain and lacking appropriate social and statutory protection (Keune, 2011; Doellgast et al., 2018; Kalleberg, 2018; Keune and Pedacci, 2019). The second phenomenon is the emergence of a platform economy where work is directly mediated or indirectly led by digital platforms (De Stefano, 2015; Irani, 2015; Stewart and Stanford, 2017; van Doorn, 2017; Gandini, 2019). Despite the literature's increased attention to the role of platform work in the labor markets, a systematic analytical approach to reasons and dimensions of precarity in platform work has not yet been established.

This paper applies the existing conceptualization of precarity to specific characteristics of on-demand platform work to respond to two research questions. First, it seeks to identify particular dimensions of precarity in on-demand platform work. Second, the paper engages in a debate on the implications of precarity in platform work on broader efforts at mitigating precarity in the labor market and on the transformation of labor market institutions such as workers' protection and interest representation. Seeking to draw implications of platform work for overcoming precarity and for transforming labor market institutions, the paper selectively focuses on on-demand platform work that embraces customer-driven tasks and can be localized in a concrete geographical, economic, social, and political setting—the type of platform economy that is associated with mobile labor markets (Codagnone and Martens, 2016; Will-Zocholl, 2017). These include both work in labor and capital platforms, such as taxi transport services (Uber and Taxify), and work related to short-term flat rentals (Airbnb), respectively. The framework seeks empirical underpinning in two countries in Central Eastern Europe—Hungary and Slovakia. While both countries demonstrate general weaknesses in enforcement of employment regulation, and the capacities of labor market institutions are modest and further eroding (Ost, 2009; Bohle and Greskovits, 2012; Doellgast et al., 2018; Trif et al., submitted), they differ in their policy responses to regulate work in the platform economy (Meszmann, 2018; Sedláková, 2018).

The contribution of the paper is two-fold. First, it sharpens and redefines the framework on work precarity by incorporating newer types of precarious work arrangements in digitalized labor markets. Second, based on original empirical evidence, it evaluates the interaction of the specificities of on-demand platform work with broader developments in labor market institutions in two Central and Eastern European (CEE) countries. Whereas, platform work in Hungary and Slovakia still appears as marginal in labor participation—compared, e.g., to Western EU member states (Eurofound, 2018; Piasna and Drahokoupil, 2019), the intensity of changes and their effects on labor market institutions are expected to differ due to different policy approaches in these regulated neoliberal market economies (Bohle and Greskovits, 2012).

In turn, understanding precarity in platform work in the empirical conditions of CEE then helps in developing the paper's main argument that while digitalization blurs labor market dualization between standard and precarious workers (cf. Rueda, 2006; Palier and Thelen, 2010; Schwander, 2018), it reinforces the existing institutional weaknesses in CEE labor markets (Ost, 2009; Bohle and Greskovits, 2012; Trif et al., submitted). The paper shows that in the case of on-demand platform work, precarity is especially pronounced in the dimension of autonomy at work and interest representation. It argues that the reason why on-demand platform work is not explicitly exposed to pressures for decreasing precarity lies neither in cases when platform work coexists with work in the traditional economy nor in cases when platform work is isolated from the traditional economy. In the former case, on-demand platform workers are able to claim social rights and job security from their jobs in the traditional economy, while in the latter case, full-time platform workers are labor market outsiders without sufficient means to organize and to influence labor market institutions. This explains the lack of pressure from platform workers to improve their labor market situation in a non-transparent regulatory framework and the lack of attention from interest representation organizations. At the same time, the coexistence of on-demand platform work with traditional jobs also blurs the lines of the established divide between labor market insiders and outsiders as distinct groups of workers (cf. Rueda, 2006; Palier and Thelen, 2010).

The paper is structured as follows. The first section conceptualizes precarity in on-demand platform work using a multidimensional understanding of precarity. The second section provides contextual information on the rise of work precarity, governing institutions of labor markets, and the status of on-demand platform workers in Hungary and Slovakia. The third section presents empirical evidence demonstrating which dimensions of precarity are found in on-demand platform work in Hungary and Slovakia and which reasons drive this precarity in different types of platform work. In turn, this evidence feeds into the concluding exploratory discussion on the impact of precarity in platform work onto a broader reconfiguration of precarity and related labor market institutions in the concluding section.

## A Multidimensional Conceptualization of Work Precarity Adapted to On-Demand Platform Work

To identify how different types of platform work differ in their extent and type of precarity and how this informs the impact of platform work on the overall reconfiguration of work precarity and related labor market institutions, the first step is to conceptualize precarity in platform work.

Platform work, or work for digital platforms, belongs to the newest phenomena facilitated by digital technologies (Akgüç et al., 2018; Berg et al., 2018). Digital platforms facilitate work arrangements where the relationship between the worker and a consumer is established via a digital market intermediary acting as a shadow employer (Friedman, 2014; Schor, 2016; Gandini, 2019). Platform work has been categorized along several dimensions. A distinction has been made between labor and capital platforms, and divisions exist also within the labor platforms. These include (a) crowd work or “click-work” platforms where workers are hired for digital-based micro-tasks (De Stefano, 2015; Gandini, 2019), (b) platforms facilitating the meeting of workers with their clients for manual labor (Heeks, 2017; Gandini, 2019), and (c) work-on-demand, or consumer-led service work including deliveries or driving where the job is organized through online platforms that retain control over important aspects of the work (De Stefano, 2015; Heeks, 2017; Stewart and Stanford, 2017; Gandini, 2019). In addition, “capital platforms,” which facilitate rent of assets, also require the application of productive labor (Stewart and Stanford, 2017). It often covers highly precarious, sometimes unpaid, types of domestic work including cleaning, maintenance, and other related services.

While crowdsourcing platforms cover predominantly online work without demonstrating geographically localized features of work, for all other types of platform work, “the local embeddedness of work and workers remain significant” (Will-Zocholl, 2017, p. 63). This is because workers meet with their clients or deliver services for them in a specific location. In turn, this paper excludes crowd work platforms from the analysis and focuses on the visible work arrangements of on-demand work, which are potentially in the scope of collective regulation by employers and trade unions (De Stefano, 2015). Thus, the paper's empirical focus is exclusively related to on-demand platform work represented by the case studies of taxi driving services (Uber/Taxify) and home rentals with associated cleaning and maintenance services (Airbnb). Such work is visible to other segments of the labor market. Despite the limited overall size of the on-demand platform economy, there is a strong qualitative pressure on regulation of platform-based work that bears implications also for labor market institutions and employment standards in general. That is, in on-demand platform work, locally embedded “broader” employment standards both inform and influence the very workers who perform these jobs, but these jobs also have a practical significance for local industrial relations and employment protection regulation.

Defining the paper's focus on on-demand platform work is instrumental to adapting the existing conceptualizations of work precarity to the specificities of this type of work arrangement. The established definition of precarious work is derived from a benchmark definition of standard employment relationship (SER). Aust and Holst (2006) define SER as a socially secured, long-term, and full-time employment with a wage that allows for a decent living. In contrast, non-standard, or atypical, work refers to the notion of a contingent workforce (cf. Heery, 2009) and involves temporary, fixed-term, part-time work, temporary agency work, and dependent self-employment (Trif et al., 2016). Kalleberg (2009, p. 2) defined precarious employment as “employment that is uncertain, unpredictable, and risky from the point of view of the worker.” While precarity is often associated with atypical work, the two are not necessarily the same (Keller and Seifert, 2013). For example, part-time employment often results from a conscious choice of an individual employee. At the same time, some standard full-time employment relationships may be precarious, e.g., because of low pay, an excessive amount of unpaid overtime, or constrained social rights and entitlements of the concerned employee.

Workers performing on-demand platform work are likely to be exposed to precarity because of their irregular work schedules and fragmented employment trajectories that are driven by fluctuations in demand for their services (Stewart and Stanford, 2017; Drahokoupil and Piasna, 2019). In addition, potential sources of precarity derive from reduced access to benefits and social security, confusion around tax issues and administrative requirements for platform workers as “service-providing individuals,” isolation and lack of interaction with co-workers, lack of on-the-job training regarding health and safety and other issues, significant occupational stress, increased workload and time pressure, and comparatively lower average net earnings than in the traditional economy (e.g., Fidler, 2016; Garben, 2017; Huws et al., 2017).

Despite evidence on exposure to precarity, a conceptualization of dimensions of precarity [e.g., (International Labour Organization (ILO), 2016), p. 19–20; (Trif et al., 2016)], specifically tailored to the unique characteristics of on-demand platform work, has not yet been established. Such a conceptualization is essential, because precarity derives not only from the type of one's employment contracts but also from seemingly invisible working conditions. In turn, workers in seemingly stable jobs may face precarity due to rising work intensity, increasing workload, work-related stress, and exposure to low pay (Pulignano et al., 2016; Grimshaw et al., 2018). In addition, precarity in on-demand work via digital platforms may be hidden in the distinct form of managerial control and the use of feedback, ranking, and rating systems that are embedded in platform work (e.g., Gandini, 2019).

Dörre (2005) identifies precarity in three spheres of work, which are well-applicable also to on-demand platform work. First, these include precarity in the material sphere, because precarious jobs do not secure decent living and job security (economic rights). Second, precarity relates to the sphere of social communication, because precarious workers are excluded from social networks at their workplace. Finally, there is a legal/institutional sphere of precarity because precarious employees are often excluded from access to certain social rights. Taken together, precarity in platform work needs to be analyzed in a multidimensional framework that acknowledges, on the one hand, how this type of work arrangement is formally anchored in the relevant labor legislation and, on the other hand, how precarity applies to particular working conditions (cf. Keller and Seifert, 2013).

First, the formal status of on-demand workers on the labor market is a source of precarity because platform work is not regulated *per se* by labor law and/or labor codes but by labor legislation governing work arrangements beyond an employment relationship. This is because most platforms differ from real employers in not recognizing workers as employees in the traditional sense (cf. Berg et al., 2018). Instead, platforms usually require for workers to take over responsibility regarding compliance with regulations and adopt a status of self-employed or individual contractor. In turn, in platform work, the concept of wages does not apply if there is no employment relationship. Instead, the concept of *income*, as explained below, is more feasible. Second, the need for customer-driven flexibility in on-demand platform work raises questions about autonomy at work. Embedding autonomy of work within the notions of supervision, control, and access to training and information, on-demand platform work is consistently identified as precarious (Pichault and McKeown, 2019). Autonomy at work is thus broader than the role of the labor market status of platform workers: it relates to unstable work schedules and highly personalized, even emotional, perceptions of precarity based on lacking access to career development and training, an information deficit, but also exposure to stress and to a metric customer evaluation (Leighton and Wynn, 2011; Deakin, 2013; Gandini, 2019; Pichault and McKeown, 2019). Taken together, the above considerations inform the multidimensional conceptualization of precarity in platform work where the six dimensions are identified.

1. **Income:** This dimension of precarity relates to the incidence of low income identified as income below two-thirds of median gross hourly wages. The concept of income captures the fact that on-demand platform workers often work on service contracts not regulated by relevant labor codes and are thus formally not in an employment relationship with wage entitlements.
2. **Job security:** Along this dimension, precarity refers to lower job security as in an SER, i.e., in terms of flexible work arrangements, seasonal fluctuations in work and fluctuations directly derived from customer ratings and evaluation systems by the platform, and lack of employment protection in case of firing.
3. **Social security:** Precarity derives from limited or no social security entitlements, including constrained holiday and collective benefit entitlements, depending on the specificities of work arrangements (small contracts, zero hours, self-employment, and similar).
4. **Working time:** Precarity derives from unpredictable working hours and overall working time, meaning also excessive and often unpaid overtime.
5. **Autonomy at work:** Precarity may originate from the lack of appropriate working conditions including limited access to training and skill development, lack of career opportunities, greater exposure to work-related stress, lack of information, and exposure to immediate feedback, ranking, and rating systems of platform workers' work from their service users/.
6. **Collective interest representation:** Precarity in this dimension originates from limited access to interest representation. This derives, first, from the lack of interest of traditional interest representation organizations to focus on platform workers and, second, from the character of platform work where workers have little opportunity to interact with each other and thus lack enabling conditions to raise their collective identity and articulate their interests.

The above conceptualization serves as an analytical tool for an assessment of dimensions of precarity in on-demand platform work in the next section. The empirical exercise focuses on two types of (a) on-demand platform work in Hungary and Slovakia: taxi/drivers via Uber and (b) workers delivering microwork, cleaning or other maintenance tasks related to property rentals via Airbnb. Evidence originates from two research projects where the authors participated: one on precarious work (PRECARIR^1^, 2014–2016) and one on platform work (IRSDACE^2^, 2016–2018). Both projects paid particular attention to monitoring and evaluating practical issues, problems, and trends related to the nature of work in given legislative, economic, and employment policy frameworks, as well as collective interest representation conditions. Data collection and analysis relied on a combination of qualitative and quantitative methods. Quantitative evidence on the character of platform work has been collected via a survey among platform workers in both countries. Qualitative evidence on the character of work and the extent of precarity has been collected via interviews with workers as well as interest representation organizations including trade unions, employer organizations, and other stakeholders, practitioners, and experts. The IRSDACE project also embraced individual and focus group interviews with platform workers. These interviews addressed the demographic and social background of on-demand platform workers, description of their working conditions and income, perceived advantages and disadvantages compared to standard employment, but also the workers' knowledge about their rights and opportunities for collective interest representation. In total, 26 interviews were conducted in Hungary and 21 interviews in Slovakia. All interviews were face-to-face, conducted by the authors in the local language, recorded, and transcribed. Before analyzing these interview data to identify precarity in platform work, the next section briefly accounts for the embeddedness of on-demand platform work in the context of Hungarian and Slovak labor markets and legislation.

## Labor Market Institutions and the Status of On-Demand Platform Workers

Hungary and Slovakia underwent a transition from state socialism to democracy and a market economy since the early 1990s. Legacies during the transition period account for important similarities in their labor market institutions, including weak law enforcement, declining trade union density and collective bargaining coverage, low levels of legally stipulated employment protection, and trends of labor market liberalization upon joining the EU. Nevertheless, there is a degree of variation in institutional regimes that are affected by particular national traditions (Bohle and Greskovits, 2012). Whereas, the significance of both national and sectoral social dialogue eroded in Hungary, along with the regulatory strength or relevance of intermediary organizations, in Slovakia, due to the existence of sector-level social dialogue and bargaining in many sectors (including transportation and tourism), social partners could potentially exert greater influence.

After 2008, both Hungary and Slovakia faced a rise in precarious work in the traditional economy via an increased use of temporary agency work, fixed-term contracts, and service contracts (Kahancová, 2016; Meszmann, 2016). In addition, low incomes push many workers to seek additional work via household work, family support work, or platform work. Such work may be on the edge of informal employment, as “invisible workers” are sometimes employed without a legal status and therefore do not have social security entitlements or access to interest representation (Kelemen, 2013; Fleck et al., 2017; Meszmann, 2018).

The rise of flexibility and precarity in the traditional economy is accompanied by the rise of platform work. Survey evidence on platform workers suggests that this form of work is overall still marginal compared to Western Europe (Drahokoupil and Piasna, 2019). Nevertheless, on-demand platform work is more widespread in Hungary than in Slovakia and localized in the capital cities Budapest and Bratislava.

The labor market status of locally embedded on-demand platform workers is informed by specific national employment legislation. In both countries, legislation does not recognize a specific category of platform work and does not offer targeted regulation acknowledging the specificities of platform work. Moreover, platform workers received marginal attention by trade unions in both countries. This fact, together with the fact that digital platforms do not consider themselves as employers (except for Uber in Slovakia, which joined the National Union of Employers), contributed to the fact that platform workers mostly have less formal, non-standard work arrangements. In Hungary, the most widespread form of on-demand platform work is bogus self-employment where most interview respondents described on-demand platform work as an entrepreneurial or “service” activity and a temporary arrangement overlapping with a swift or sometimes desperate need to find an income-generating activity. Especially for younger workers and foreigners, it overlapped with their entry into the Hungarian labor market. Similarly, in Slovakia, self-employment is also a common labor market status for on-demand platform workers. Since the majority of Slovak interview respondents justified their platform work as a source of additional income, their employment status is usually a combination of several labor market statuses, for instance, solo self-employment combined with a standard employment contract or service contracts (Sedláková, 2018). This situation may change for platform taxi drivers after the 2019 legislative changes to road transport legislation, which re-regulates the taxi service provision including the service via online platforms and facilitates a greater use of standard employment contracts. In platform work in accommodation services, Airbnb providers utilize the advantage of a gray zone between two labor market statuses: a natural person offering short-term rentals with a local tax payment obligation and a licensed provider of accommodation services (including additional services such as cleaning) according to the Trade Licensing Act (Sedláková, 2018). In addition, students are often hired as seasonal cleaners without an official contract. Cash payments, which are also common for microwork and undeclared or under-declared household work, were also reported in the interviews.

In sum, in the conditions of weak law enforcement and weak interest representation, digital platforms facilitating on-demand work possess great discretion over defining the labor market status of their workers. This includes shifting all risks and formal obligations related to licensing and tax issues onto the workers. Nevertheless, most of our interviewed on-demand platform workers, especially in Slovakia, had their first jobs in traditional sectors or were students. This means that their labor market status was not predominantly defined through their platform work. In turn, they did not highlight the volatility of on-demand platform work *per se*.

## Precarity in On-Demand Platform Work

The status of on-demand platform workers on the Hungarian and Slovak labor markets suggests distinct features that may qualify on-demand platform work in itself as precarious. This section goes deeper and identifies the sources of precarity in on-demand platform work in Hungary and Slovakia across the six dimensions of precarity presented earlier. Attention is also paid to differences between two broadly defined on-demand platform sectors: taxi driving for Uber and Taxify and work related to property rental services via Airbnb. Table 1 presents a qualitative–comparative evaluation of precarity according to six dimensions.

**Table 1:** Assessment of precarity in six dimensions.

**Income**	**Working time**	**Autonomy at work**	**Job security**	**Social security**	**Representation**
Minimum wage not applicable Variation regarding discretion over pay setting: more price-setting coordination (taxi services) vs. direct exposure to self-exploiting competition (e.g., in price setting for cleaning, guest reception, maintenance, etc.) Hidden costs of on-demand platform work (e.g., investments in work tools)	High variation with potential high risk. Irregular working hours, difficult reconciliation with other activities, and at the same time more flexibility	Low Exposure to direct evaluation by customers with direct impact on expected demand for services and thus income level Lack of information on requirements and on-the-job training rights	Medium Worker-driven, pressure to work more to increase income, indirect dependence on customer ratings, flexibility in accommodation sector	Low (informal employment) or medium if self-employed status or if there is a combination with other jobs (or student status, retired, etc.)	Low (accommodation service and transport in Hungary) or non-existent (Slovakia, microworkers in Hungary) Marginal access to collective interest representation; workers do not engage in social interaction with co-workers or only virtually

*Authors' assessment based on original empirical evidence (on-demand platform work in Hungary and Slovakia)*.

### Income

The income of on-demand platform workers likely differs between the two sectors. Whereas, in on-demand taxi services, income is usually based on a well-defined, set rate with less discretion over pay setting, in accommodation services, the income generated by apartment rent hides the labor part behind it (reception, cleaning) that thus might be a hotbed of (self-)exploitation. In both cases, there is a radical exposure of workers to risks of market demand, with a varying level of autonomy to calculate the benchmark of a decent income. Whereas, in taxi services, the market demand is, in general, high in both countries, in accommodation, such as cleaning, it is lower. Especially in Slovakia, the demand for Airbnb and associated services in the capital city (with the highest concentration of apartment renting) is not comparable to that in Budapest. Thus, it can function only as an additional, supplementary income.

For microworkers, especially in Slovakia, remuneration was not seen as fair, especially when pushed down by inexperienced, unqualified, and/or younger workers (students, but even migrant workers). In personal transport of both countries, taxi drivers have no control over their pay, which is set by the platform, and drivers have no negotiating powers over it. In Slovakia, this fact was cited as one of the barriers to full-time employment in the sector (Sedláková, 2018). In Hungary, taxi drivers responded with quantitative flexibility, 10–12 h of work, in order to increase the likelihood of generating sufficient income. On the contrary, Airbnb hosts set their income for accommodation themselves. Yet, this autonomy could backfire, as shown in the example of Hungary: there was a constant complaint that there was a “selling at any price” logic present among Airbnb hosts, which further exerts a downward pressure on income levels.

In Hungary, interview respondents confirmed that hourly income in on-demand platform work is either significantly higher than in other jobs across CEE or more difficult to calculate due to hidden costs related to investments, maintenance, etc. This is why compared to other countries, in Hungary and Slovakia, calls for minimum wages applicable to platform work are scarce, both from workers themselves and from trade unions.

In sum, on the dimension of income, on-demand platform work cannot be classified as precarious, but for full-time platform workers, the risk of precarity is quite high. As long as platform work generates secondary income, as most of the cases especially in Slovakia show, workers may be less concerned about low income levels from their platform work and more concerned about competition with other service providers and maximizing their individual income. On the negative end, non-transparent income levels hide precarity on the income dimension, and apart from increasing the workload, there are no available mechanisms to positively influence the workers' own income levels—a finding that we will deal with more in the interest representation dimension.

### Working Time

Precarity in on-demand platform work is manifested in unpredictable working hours (in the case of the accommodation sector) and length of working time (more in taxi transport). Nevertheless, most interview and survey respondents in both countries identified flexibility of working time as the major advantage of platform work. This seemed to be the case especially among those who had defined, or could clearly define, their working time, and for those who were working as on-demand workforce on a part-time basis. However, after detailed scrutiny, irregular working hours and difficult reconciliation with other, more stable jobs and family time became the critical aspect of this “not-so-flexible flexibility.” Especially in accommodation services, a push for a constant availability, enhanced by the exposure to direct customer ratings, motivates workers to be available at virtually any time.

Flexibility in working time appears to be a central dimension in unpacking precarity as it taps back into income levels, job security, and especially autonomy at work—due to fear and implications of negative ratings. For platform-driven taxi drivers in both countries, working extended long hours was voluntary, but it raised health and safety issues. In providing accommodation services and associated microwork, one had to know how to manage and organize one's own time in order to come to terms with high variations and daily rhythms of on-demand work. The more informed and skillful workers translated extra, non-regular work hours to extra benefits or fees from customers. For those who were unprepared for this fluctuation, undefined working time caused high stress. Hence, the source of precarity in working time arrangements was especially interconnected with autonomy at work, related to unpreparedness, lack of power, information, or autonomy to definite or limit one's own working time for on-demand work (see below), and risks of self-exploitative practices.

### Autonomy at Work

Lack of information, concerns over liability, and health and safety of on-demand platform workers came up as strongly voiced concerns. Interviewed platform workers were typically unsure what happens in case of accidents or problems. Most full-time platform worker interviewees in Hungary listed the lack of information and training for the job as the most problematic dimension. This was not the case in Slovakia, where the issues of professional training were voiced only in relation to taxi services. Interviewees mentioned lack of information on rules of taxation and administration for novices and lack of training for the job, with the necessary skills in communication, conflict management, and problem solving, especially in Hungary (Meszmann, 2018). Although not explicitly, similar concerns were recorded among taxi drivers in Slovakia, who often worried about the background of the workers and state of their professional knowledge (Sedláková, 2018). Last but not least, some Hungarian platform workers felt that their economic activity enjoyed a very low social prestige. Moreover, they also felt that conflicts that platforms generated in the broader society translated into an unfavorable, unsupportive social environment vis-à-vis on-demand platform workers.

### Job Security

The dimension of job security is particularly obscure in on-demand platform work. From a legal perspective, job security of platform workers is very low. This is due to widespread operation of platform workers as independent contractors or economically dependent workers (bogus civil law contractees), or even undeclared workers (especially apartment cleaners). Nevertheless, the interviewed platform workers across both studied types of on-demand work experienced their job security as moderate and depending mostly on the (seasonal) business cycle, as well as on insecurities stemming from a changing regulation of the sector. In Hungary, the character of on-demand cleaning jobs as platform-mediated accommodation services was considered to be temporary only, due to its seasonal character. In taxi transport of both countries, the line between platform work and traditional taxi driving was less pronounced, given that both groups, platform-based taxi services and traditional taxi providers, introduced software applications. Thus, in taxi transport in both countries, job security was significantly higher.

Despite dependency on customer rankings and ratings, neither of the interviewees except one spelled this out as a major factor causing lower job security. On the other hand, indirectly, the demand for flexible work and adaptation to a changing regulatory environment caused, at times, higher rates of quitting the platform, especially among platform workers providing accommodation services. As aptly formulated by one respondent providing accommodation-related services, in the triangle between the (intermediary) employer, customer, and worker, the last had the weakest position, without a real voice. Due to the seasonal character of on-demand work, or its combination with other forms of employment, job security was not judged as problematic.

In sum, job security is low to moderate in on-demand platform work. There is a strong influence of seasonality and a more obscure effect of customer ratings. Interactions with other forms of work in the economy again demonstrate the importance of assessing precarity in on-demand platform work only in the context of workers' overall labor market positions.

### Social Security

Social security of platform workers appears to be dependent on their labor market status and can thus range from highly precarious (as in informal economy) to low-standard, in cases of part-time platform work, when a social security arrangement was gained from the main job. In Hungary, platform workers work at best under civil law relationship-based service contracts and thus do not enjoy all elements of social protection. Except equal treatment, free movement, and access to social security services, other elements of social protection are thus not available to the self-employed, such as paid leaves, redundancies, family benefits, etc. Moreover, the work of individual entrepreneurs is not considered “organized work”; thus they do not fall under the Hungarian Health and Safety Act. Simplified employment for seasonal work is based on the labor code, but provisions on unpaid leave, sick leave, etc. do not apply. However, in the case of self-employment, irrespectively of the increasingly flexible income threshold, the beneficial taxation scheme also translates into a default of low long-term social security, i.e., extremely low retirement savings. Likewise, due to no specific regulation of platform work on the labor market in Slovakia, platform workers have no specific entitlements for social protection. In addition, based on the evidence from the interviews, platform workers do not consider this aspect as problematic and rarely think about the consequences of it, unless platform work becomes their main source of income.

### Interest Representation

Platform workers in both countries are neither associated with nor represented by trade unions. Microworkers as well as individual entrepreneurs in Hungary fulfill the criteria for membership with some civil and interest-based associations but do not fulfill the established criteria to become trade union members. Moreover, workers are even more atomized than in traditional sectors, and the possibility of interest articulation via trade unions or alternative organizations is typically not recognized. In Slovakia, the discussion among the traditional trade unions of whether to include platform workers into their structures was not even raised yet, and trade unionists point out the structural obstacles within their traditional organizations for the new types of workers to join the unions (Sedláková, 2018). Most interviewees in Hungary and Slovakia were not aware of trade unions or of any associations that could provide useful information, let alone serve as an agency of their interest representation. This situation is reinforced by the fact that those working or providing the bulk of labor in these sectors come from social groups which typically provide the most precarious work in patriarchal and closed clientelist societies, including youth, women, and immigrants.

In Hungary, there was only one association that approximated an interest representative organization of on-demand platform workers, the association of small, individual accommodation providers (MAKE—“Magyar ApartmanKiadók Egyesület”—Association of apartment renters). However, the association was first of all gathering individual owners of apartments and acted as their voice for pressing local government for a low threshold of regulation—and it was not associated with labor that came with renting out apartments. Nevertheless, it provided useful information and training to its members and filled an important gap in raising awareness in platform workers' voice. Similar attempts have been recorded in Slovakia, where the civic association “Vitaj Doma” (Welcome Home) was formed by the owners and providers of Airbnb services. The organization, whose functioning remains mostly visible via discussions on social networks, functions rather as a forum offering information on legal changes, tax requirements, and vacant jobs (mostly in cleaning).

### Summary

Considering the presented dimensions of precarity, we conclude that the risk and source of precarity in on-demand platform work does not come from low income or irregular working time but is especially manifest in lacking autonomy at work and collective interest representation. On all other dimensions, precarity depends on sector-specific regulation and hidden risks, e.g., consumer rating for income or job security, or net incomes given the costs of engagement in the activity.

The working time of on-demand workers indeed turns into a burden when reconciling work with family and leisure. It creates challenges for coordinating working times in the case of multiple jobs and pushes workers to be available around the clock and respond to customers' queries quickly. This connects with precarity in autonomy at work: on-demand platform workers are often not informed about requirements and (a lack of) their rights and may experience administrative, market demand risks and customer ratings only when already engaged in on-demand platform work. Finally, on-demand platform workers have limited to no access to interest representation due to little contact with other workers and an unclear formal status of the platform worker on the labor market (between a worker, an entrepreneur, a freelancer, and even an owner of capital in the case of accommodation), which structures access to established institution representation channels in traditional sectors of the economy.

## Discussion

Uncovering the sources of precarity in on-demand platform work fuels a discussion on how such precarity in new forms of work contributes to reconfiguration of a general understanding of precarity and related employment regulation within broader labor market conditions. Evidence in this paper allows identifying four factors that are crucial in understanding how digitalization facilitates, or even reinforces, precarity and has direct implications for labor market segmentation and broader labor market institutions. These factors include:

– Complementarities between platform work and work in the traditional economy
– A delayed regulatory response to precarity in platform work
– Crisis of interest representation of platform workers via established structures
– Scope of the platform economy within the entire economy.

### Complementarity Between Platform Work and Work in the Traditional Economy

Evidence reveals that platform work in Hungary and Slovakia is perceived by most platform workers as an additional source of income but not their institutional anchor to the labor market. For the majority of platform workers, their on-demand platform work is secondary or serves as an entry point for labor market outsiders (especially in Hungary). The structure of platform workers, their overall labor market situation, and the structure of their income are thus central in understanding why precarity in on-demand platform work persists. The presented evidence allows us to frame the argument on persistence of precarity in the platform economy through its coexistence with working conditions in the traditional economy (see Table 2).

**Table 2:** Coexistence between traditional and platform work as a factor in explaining precarity.

		**Platform work**
Traditional work	Yes	Workers willing to accept precarious on-demand platform work because of drawing their social rights from traditional work. Explains lack of initiatives to decrease precarity in platform work.
	No	Workers in on-demand platform work would possibly be motivated to decrease precarity but lack access to job security, decent income, and interest representation in the traditional economy. Their weakness to organize to mitigate precarity explains its persistence.

*Authors' elaboration*.

Based on Table 2, we argue that if on-demand platform workers simultaneously have a non-precarious, stable job and predictable income in traditional economic sectors, their social rights, job security, and access to interest representation is secured. Therefore, platform work, with its inherently precarious character, is not exposed to pressures to improve its dimensions of precarity. And even if the primary jobs of platform workers are precarious in the traditional economic sectors, there is a lack of pressure from workers to improve their situation due to lacking access to interest representation, or voice, both in the traditional and in the platform economy. Precarious forms of platform work thus enjoy stability in their coexistence with other forms of employment in Hungarian and Slovak economies.

### Delayed Regulatory Response to Working Conditions in On-Demand Platform Work

As outlined above, understanding the nature of work in the platform economy is only possible when analyzing platform work as part of the overall system where the traditional and the platform economy coexist. Regulation of employment conditions in Hungary and Slovakia has been subject to adjustment and stabilization in the past three decades since both countries embarked on a transition to democracy and a market economy. Employment legislation as well as the structures of interest representation and collective bargaining have thus evolved and stabilized prior to the 2008 crisis. Still, the post-crisis period resembled a modest shock to this system in both countries. While employment levels quickly recovered in the private sector despite an initial decrease in production and in the public sector despite austerity measures, a crisis in employment regulation served as a factor enabling the rise of other forms of precarious work including platform work (Srnicek, 2017).

The legislative response to precarious work in the traditional economy after the crisis is now repeated in response to a platform economy. Meanwhile, during the period of a regulatory gap, work precarity in the platform economy has been further embedding in order to embody the currently typical features of on-demand platform work. One of the few examples of the regulatory responses is recent legislative changes establishing the same professional requirements for drivers working in traditional taxi services as well as drivers working for platforms like Uber and Bolt. This regulation facilitates convergence in working conditions between traditional sectors and the platform economy, but the criterion of reducing precarity has not yet been met due to too few similar regulatory initiatives. In addition, attempts to regulate the platform economy focus on operational aspects of platforms at the national level while leaving the responsibility to mitigate precarity in the workers' hands.

### Crisis of Interest Representation

The wider effect of precarity relates to the fact that it may create a large group of vulnerable employees detached from the rest of the labor market participants and society (cf. Dörre, 2005). Nevertheless, this paper shows that in fact, workers in standard jobs and on-demand platform workers may be the same persons, because on-demand platform work is often used as a second job next to a job with a more stable labor market status. Thus, the dividing lines between standard and precarious workers are blurred. Since on-demand workers using platforms as their second job have access to social rights and employment protection through their standard jobs, they do not seek ways to decrease precarity in their platform work. On the opposite spectrum are full-time platform workers, who are true labor market outsiders without social protection and job security, with little autonomy at work, lack of access to interest representation, and high exposure to risks. Nevertheless, pressures to decrease precarity do not come from this group either, because of their overall weakness to organize and voice their interests. The blurred line between standard and precarious platform workers also has implications for interest representation. Employee representatives not only face a challenge to represent two groups of workers with different interests from among the standard and the contingent workforce but also need to address different interests embodied in the same group of workers who are simultaneously in precarious and non-precarious labor market positions. In fact, trade unions' attention to the interests of platform workers is marginal, and they do not exert any significant pressure to reduce their precarity.

Despite the weakness of on-demand platform workers to organize, the authors' focus group interviews with platform workers in Hungary and Slovakia revealed their potential interest to organize and seek collective interest representation in the future. In Slovakia, Uber joined the peak-level employers' association National Union of Employers (RUZ) in April 2018, which may give additional impetus for trade unions to seek representation of platform workers. Still, in general, the paper shows that a precondition for mobilization of on-demand platform workers is a growing size of the platform economy, further deterioration of working conditions that would mobilize workers to be more attentive to their social rights, and also changes on the side of established trade unions in their willingness to represent platform workers. As long as platform work is treated merely as an additional source of income, greater mobilization both from trade unions as well as from the side of workers is not expected.

### Scope of Platform Work Within the Labor Market

Finally, the size of the on-demand platform economy and its coexistence with work arrangements in the traditional economy are crucial in understanding the impact of the platform economy on the general framing of precarity in the labor market and related labor market institutions. We have shown in this paper that the platform economy in Hungary and Slovakia still consists of a marginal source of the population's income and labor market attachment. Nevertheless, there are signals from neighboring countries, e.g., Czechia, but already partly in Hungary, that a growing demand for Airbnb housing services further deepens precarity especially in the dimensions of working time and autonomy at work due to stress exposure and flexibility in cleaning jobs. As demand will increase for platform-mediated housing and transport services, the share of on-demand platform work is expected to grow. In that case, the analysis presented in this paper needs to be revisited with updated analytical tools and empirical evidence.

## Data Availability Statement

All datasets generated for this study are included in the article/supplementary material.

## Author Contributions

MK, TM, and MS contributed with equal share to the original research piece by discussing its focus, evidence and scientific contribution. They worked together in the two research projects where data for this paper have been collected. In terms of writing, the authors contributed the following way: MK—introduction, conceptual framework, discussion and conclusions, overall review. TM—introduction, evidence on precarious work (section: Labour Market Institutions and the Status of On-Demand Platform Workers), and evaluation of platform work (section: Precarity in On-Demand Platform Work), overall review. MS—evidence on precarious work (section: Labour Market Institutions and the Status of On-Demand Platform Workers) and evaluation of platform work (section: Precarity in On-Demand Platform Work), overall review, assembling the final version and formatting the article.

### Conflict of Interest

The authors declare that the research was conducted in the absence of any commercial or financial relationships that could be construed as a potential conflict of interest.

## Footnotes

^1^https://www.dcu.ie/link/current-projects/precarir2014-2016.shtml

^2^https://www.ceps.eu/ceps-projects/industrial-relations-and-social-dialogue-in-the-age-of-collaborative-economy-irsdace/

## References

[B1] Akgüç M. Beblavý M. Cirule E. Kilhoffer Z. (2018). Industrial Relations and Social Dialogue in the Age of Collaborative Economy (IRSDACE) Comparative Report. Brussels: CEPS Research Paper. Available online at: https://www.ceps.eu/ceps-publications/industrial-relations-and-social-dialogue-age-collaborative-economy-comparative-report/

[B2] Aust A. Holst H. (2006). Von der ignoranz zur organisierung? Gewerkschaftliche Strategien im Umgang mit atypisch Beschäftigten am Beispiel von Callcentern und Leiharbeit. [From ignorance to organizing? Trade union strategies towards atypical workers in the example of call centres and temporary agency work]. Indus. Beziehungen 13, 291–313. Available online at: https://nbn-resolving.org/urn:nbn:de:0168-ssoar-343004

[B3] Berg J. Furrer M. Harmon E. Rani U. Six Silberman M. (2018). Digital Platforms and the Future of Work: Towards Decent Work in the Online Work. Geneva: ILO.

[B4] Bohle D. Greskovits B. (2012). Capitalist Diversity on Europe's Periphery. Ithaca, NY: Cornell University Press.

[B5] Codagnone C. Martens B. (2016). Scoping the Sharing Economy: Origins, Definitions, Impact and Regulatory Issues. Institute for Prospective Technological Studies Digital Economy Working Paper. Available online at: https://ec.europa.eu/jrc/sites/jrcsh/files/JRC100369.pdf (accessed August 9, 2019).

[B6] De Stefano V. (2015). The Rise of the ‘Just-in-Time Workforce': On-Demand Work, Crowdwork and Labour Protection in the ‘Gig Economy'. Conditions of Work and Employment Series 71. Geneva: ILO.

[B7] Deakin S. (2013). Addressing Labour Market Segmentation: The Role of Labour Law. Cambridge: Centre for Business Research, University of Cambridge.

[B8] Doellgast V. Lillie N. Pulignano V. (eds) (2018). Reconstructing Solidarity: Labour Unions, Precarious Work, and the Politics of Institutional Change in Europe. Oxford: Oxford University Press Publications. 10.1093/oso/9780198791843.001.0001

[B9] Dörre K. (2005). ‘Die Zone der Verwundbarkeit. Unsichere Beschäftigungsverhältnisse, Prekarisierung und die Gewerkschaften' [*Zone of Vulnerability: Insecure employment relations, precarization and trade unions*]. In: M. Sommer; K. Dörre and U. Schneidewind, Die Zukunft war vorgestern: der Wandel der Arbeitsverhältnisse: Unsicherheit statt Normalarbeitsverhältnis? [The future was the day before yesterday: Transformation of Employment Relations: Insecurity instead of standard employment relations?] (Oldenburg: Bibliotheks- und Informationssystem der Universität, 19–55.

[B10] Drahokoupil J. Piasna A. (2019). Digital Labour in Central and Eastern Europe: Evidence from the ETUI Internet and Platform Work Survey. ETUI Working Paper. Brussels: ETUI.

[B11] Eurofound (2018). Employment and Working Conditions of Selected Types of Platform Work. Luxembourg: Publications Office of the European Union. Available online at: https://digitalcommons.ilr.cornell.edu/cgi/viewcontent.cgi?article=1640&context=intl

[B12] Fidler D. (2016). Work, Interrupted the New Labor Economics of Platforms. IFTF Research Report. Available online at: www.iftf.org/fileadmin/user_upload/downloads/wfi/IFTF_Work-Interrupted_FullReport.pdf (accessed August 9, 2019).

[B13] Fleck Z. Kiss V. Tóth F. Neumann L. Kenéz A. Bajnok D. (2017). A Jogtudat Narratí*v Értelmezése*. Budapest: ELTE - Eötvös Kiadó.

[B14] Friedman G. (2014). Workers without employers: shadow corporations and the rise of the gig economy. Rev. Keynes. Econ. 2, 171–188. 10.4337/roke.2014.02.03

[B15] Gandini A. (2019). Labour process theory and the gig economy. Hum. Relat. 72, 1039–1056. 10.1177/0018726718790002

[B16] Garben S. (2017). Protecting Workers in the Online Platform Economy: An Overview of Regulatory and Policy Developments in the EU. European Risk Observatory Discussion Paper. EU OSHA. Available online at: https://osha.europa.eu/en/tools-and-publications/publications/regulating-occupational-safety-and-health-impact-online-platform/view

[B17] Grimshaw D. Marino S. Anxo D. Gautié J. Neumann L. Weinkopf C. (2018). “Negotiating better conditions for workers during austerity in Europe: unions' local strategies towards low pay and outsourcing in local government,” in Reconstructing Solidarity: Labour Unions, Precarious Work and the Politics of Institutional Change in Europe, eds Doellgast V. Lillie N. Pulignano V. (Oxford: Oxford University Press), 42–66. 10.1093/oso/9780198791843.003.0002

[B18] Heeks R. (2017). Decent Work and the Digital Gig Economy: A Developing Country Perspective on Employment Impacts and Standards in Online Outsourcing, Crowdwork, etc. Development Informatics Working Paper Series No. 71. 10.2139/ssrn.3431033

[B19] Heery E. (2009). Trade unions and contingent labour: scale and method. Camb. J. Reg. Econ. Soc. 2, 429–442. 10.1093/cjres/rsp020

[B20] Huws U. Spencer N. H. Syrdal D. S. Holts K. (2017). Work in the European Gig Economy: Research Results From the UK, Sweden, Germany, Austria, the Netherlands, Switzerland and Italy. Brussels: Foundation of European Progressive Studies and UNI Europa.

[B21] International Labour Organization (ILO) (2016). Non-Standard Employment Around the World: Understanding Challenges, Shaping Prospects. Geneva: ILO.

[B22] Irani L. (2015). Difference and dependence among digital workers: the case of amazon mechanical turk. South Atlant. Q. 114, 225–234. 10.1215/00382876-2831665

[B23] Kahancová M. (2016). The *Rise of the Dual Labour Market: Fighting Precarious Employment in the New Member States through Industrial Relations (PRECARIR) Country Report: Slovakia*. Research Report No. 19. Bratislava: CELSI.

[B24] Kalleberg A. L. (2009). Precarious work, insecure workers: employment relations in transition. Am. Sociol. Rev. 74, 1–22. 10.1177/000312240907400101

[B25] Kalleberg A. L. (2018). Precarious Lives: Job Insecurity and Well-Being in Rich Democracies. Cambridge: Polity Press. PMC6703155 31435077

[B26] Kelemen M. (2013). A háztartási alkalmazottak foglalkoztatásának kérdései Magyarországon - a láthatatlan munkaero [Questions related to domestic workers in Hungary – the invisible workforce]. Esély 3, 3–24. Available online at: http://www.esely.org/kiadvanyok/2013_3/kelemen.pdf

[B27] Keller B. Seifert H. (2013). Atypische Beschäftigung Zwischen Prekarität und Normalität. Entwicklung, Strukturen und Bestimmungsgründe im Überblick [Atypical Employment Between Precarity and Normality. Overview of Developments, Structures and Determining Factors]. Berlin: Edition Sigma.

[B28] Keune M. (2011). Trade union responses to precarious work in seven European countries. Int. J. Lab. Res. 5, 59–78. Available online at: https://hdl.handle.net/11245/1.398035

[B29] Keune M. Pedacci M. (2019). Trade union strategies against precarious work: common trends and sectoral divergence in the EU. Eur. J. Indust. Relat. 10.1177/0959680119827182.

[B30] Leighton P. Wynn M. (2011). Classifying employment relationships: more sliding doors or a better regulatory framework? Indus. Law J. 40, 5–44. 10.1093/indlaw/dwq02926954924

[B31] Meszmann T. T. (2016). The Rise of the Dual Labour Market: Fighting Precarious Employment in the New Member States through Industrial Relations (PRECARIR) Country Report: Hungary. Bratislava: CELSI. Research Report No. 12.

[B32] Meszmann T. T. (2018). Industrial Relations and Social Dialogue in the Age of Collaborative Economy (IRSDACE) National Report: Hungary. CELSI Research Report No. 27/2018. Available online at: https://celsi.sk/en/publications/research-reports/download/28/industrial-relations-and-social-dialogue-in-the-age-of-collaborative-economy-irsdace-national-report-hungary/

[B33] Ost D. (2009). The consequences of postcommunism: trade Unions in Eastern Europe's future. East Eur. Politics Soc. 23, 13–33. 10.1177/0888325408326791

[B34] Palier B. Thelen K. (2010). Institutionalizing dualism: complementarities and change in France and Germany. Polit. Soc. 38, 119–148. 10.1177/0032329209357888

[B35] Piasna A. Drahokoupil J. (2019). Digital labour in central and eastern Europe: evidence from the ETUI Internet and Platform Work Survey. ETUI. Available online at: https://www.etui.org/content/download/37603/377074/file/WP+2019+12++Digital+Labour+Web+version.pdf

[B36] Pichault F. McKeown T. (2019). Autonomy at work in the gig economy: analysing work status, work content and working conditions of independent professionals. New Technol. Work Employ. 34, 59–72. 10.1111/ntwe.12132

[B37] Pulignano V. Doerflinger N. De Franceschi F. (2016). Flexibility and security within European labour markets: the role of local bargaining and the “trade-offs” within multinationals' subsidiaries in Belgium, Britain and Germany. ILR Rev. 69, 605–630. 10.1177/0019793916628862

[B38] Rueda D. (2006). Social democracy and active labour-market policies: insiders, outsiders and the politics of employment protection. Br. J. Polit. Sci. 36, 385–406. 10.1017/S0007123406000214

[B39] Schor J. (2016). Debating the sharing economy. J. Self Govern. Manage. Econ. 4, 7–22. 10.22381/JSME4320161

[B40] Schwander H. (2018). Labor market dualization and insider–outsider divides: why this new conflict matters. Polit. Stud. Rev. 17, 14–29. 10.1177/1478929918790872

[B41] Sedláková M. (2018). Industrial Relations and Social Dialogue in the Age of Collaborative Economy (IRSDACE) National Report: Slovakia. Bratislava: CELSI. Research Report No. 28.

[B42] Srnicek N. (2017). The challenges of platform capitalism: understanding the logic of a new business model. Progr. Rev. 24, 254–257. 10.1111/newe.12023

[B43] Stewart A. Stanford J. (2017). Regulating work in the gig economy: what are the options? Lab. Relat. Rev. 28, 420–437. 10.1177/1035304617722461

[B44] Trif A. Koukiadaki A. Kahancová M. (2016). The Rise of the Dual Labour Market: Fighting Precarious Employment in the New Member States Through Industrial Relations (PRECARIR), Comparative Report. DCU Research Report. Available online at: https://www.dcu.ie/link/current-projects/precarir2014-2016.shtml (accessed August 9, 2019).

[B45] van Doorn N. (2017). Platform labor: on the gendered and racialized exploitation of low-income service work in the ‘on-demand' economy. Commun. Soc. 20, 898–914. 10.1080/1369118X.2017.1294194

[B46] Will-Zocholl M. (2017). “Virtual temptations: reorganising work under conditions of digitisation, virtualisation and informatisation,” in The New Digital Workplace: How New Technologies Revolutionise Work, eds Briken K. Chillas S. Krzywdzinski M. Marks A. (Palgrave: MacMillan), 62–86. 10.1057/978-1-137-61014-0_4

